# Migrant Caregivers of Older People in Spain: Qualitative Insights into Relatives’ Experiences

**DOI:** 10.3390/ijerph17082953

**Published:** 2020-04-24

**Authors:** María José Morales-Gázquez, Epifanía Natalia Medina-Artiles, Remedios López-Liria, José Manuel Aguilar-Parra, Rubén Trigueros-Ramos, Jerónimo J. González-Bernal, Patricia Rocamora-Pérez

**Affiliations:** 1 Department of Nursing, University of Las Palmas de Gran Canaria (ULPGC), Juan de Quesada, 30, 35001 Las Palmas de Gran Canaria, Spain; mariajose.morales@ulpgc.es (M.J.M.-G.); epifania.medina@ulpgc.es (E.N.M.-A.); 2 Health Research Centre, Department of Nursing, Physiotherapy and Medicine, University of Almería, Carretera del Sacramento s/n, La Cañada de San Urbano, 04120 Almería, Spain; rocamora@ual.es; 3 Department of Psychology, University of Almería, Carretera del Sacramento s/n, La Cañada de San Urbano, 04120 Almería, Spain; jmaguilar@ual.es; 4 Department of Language and Education, University of Antonio de Nebrija, 28015 Madrid, Spain; 5 Department of Psychology, University of Burgos, 09001 Burgos, Spain; jejavier@ubu.es

**Keywords:** caregiver, family, older adults, emotions, experiences, migrant

## Abstract

The traditional structure of families is undergoing profound changes, causing the so-called “crisis of family care.” This study describes the experiences and emotions of the family member who hires migrant caregivers for the older people. This is a qualitative study using a phenomenological design with nine women participants between 53 and 72 years of age. The data collection was carried out through two in-depth interviews and a focus group. There were three major topics: (1) the women in this study recognized that they were not able to take care of the family member directly, due to their responsibilities as female workers and mothers. The fact that migrant caregivers were chosen was conjunctural, where economic reasons were more important. (2) The family members supported the caregivers by teaching them about care and also resolving conflicts produced by culture shock. (3) Trusting the caregiver was a gradual process; the family members felt a complex set of emotions (insecurity, gratitude for the help, moral obligation). In conclusion, they wanted a caregiver who would provide the elder dependent with the love and compassion that they, as daughters, would provide if they had time to do so. The family became the caregiver’s managers and assumed the responsibility of training and helping them.

## 1. Introduction 

With the rise of the older population, the number of people dependent on others when performing basic activities of daily living (ADLs) increases. In line with the worldwide trend, the European population continues its progressive aging. The life expectancy of Spaniards is among the highest in Europe and the world [1,2].

Western social health systems, regulated by the Dependency Law in Spain [3], do not guarantee adequate care for people with long-term care needs [4]. In this country, the responsibility for this care has traditionally rested on the family [5,6,7,8], redefining family as “the group of people who care for dependents without seeking any direct and immediate monetary compensation” [9]. Within the family, this duty has invariably fallen upon women [10,11,12]. However, the traditional structure of families is undergoing profound changes. One of the most relevant changes is about the female of the new generation [13,14]. Women have begun to expand their world by leaving their homes to work and develop in different areas of their life, increasingly moving away from their customary duties of domestic work and caring for other members of the household [7,15,16]. This paradigm shift is occurring both in Spain and internationally, and it is causing the so-called crisis of family care, to which governments will have to respond. However, the inadequacy of the government to respond is forcing families to solve the problem. Thus, emancipated women hire other women, migrants, as a solution to the global shortage of means for long-term care [5,17,18,19,20].

Just as aging can differ from one culture to another, not only in terms of ethnic and racial differences, but also in urban and rural, socioeconomic, and environmental differences, beliefs about elder support also differ from country to country [21,22]. The Canary Islands are not an exception to this reality. They present typical socio-cultural characteristics of rural, isolated territories, where the norms are more rigid and defined. Sanctions can be imposed more easily, demonstrating excellent control over the behavior of its inhabitants, typical of small communities and neighborhoods [23,24]. The behavior of the individual here is situational and contextual, rather than of character. This culture of control behavior is through external factors more than internal ones, where the emphasis is placed on well-being and fitting into society, rather than personal and individual behavior [25]. 

At an international level, the responsibility for assuming the care of a dependent relative rests on the complexity of the political and socioeconomic contexts existing in each country [21]. The truth is that the Canarian women, like other women, share the belief that the duty of caring for the older person in the family is natural [26], due to the universal culture of female subordination to men [27]. However, the cultures of control over island women promoted by rurality and geographical isolation, makes them feel the guilt or shame more intensely if they do not comply with the cultural norm or transgress social precepts [23,24]. Moreover, although it depends on the personality and specific circumstances, the feeling of guilt appears frequently and it is more related to moral transgression than to actual damage to third parties [23,24]. In this sense, feeling guilty is strongly associated with the social pressure they feel, in a town where everyone knows each other and guilt is directly related to shame [28]. Guilt is also associated with a sense of responsibility toward the well-being of the family member in need [29]. 

Under these contexts and socio-cultural circumstances, women have a variety of reasons to care for a dependent family member, such as the desire that the family member stay at home, the love they feel toward him/her, filial or conjugal obligation, social pressure, and even the desire to set an example [30]. However, they also experience feelings of sadness, guilt, overburden, impotence, despair, anger, and fear for the future [31]. 

Immersed in this emotional and cultural world, Canarian women, like many others, were forced to accept that they could not take care of dependent older individuals [15] and thus, they hire migrant women as a solution. Actually, the shortage of people willing to work in this activity has demonstrated the need to import immigrant labor [16]. Studies in different countries have reported that the number of immigrants who care for older people is higher than that of natives [32]. 

Many families chose to hire migrant women. These women saw an opportunity to solve their unemployment issues or illegal situation by caring for older individuals, which provides them a place to live, food, a fixed salary and the ability to support their own family in their original country. The tendency of this type of caregiver to receive a lower than average wage, as well as not to require a legal contract due to their illegal situation, are the reasons why families opt for them [32,33]. 

The environment where this care is provided is home, which not only involves privacy and intimacy, but also makes the care hidden, little known, and difficult to access [5]. This study aims to bring visibility to this complex phenomenon. The process of hiring an unqualified stranger to take care of a dependent relative is sensitive, and emotions and responsibility play equally important roles. However, to date, no study has been found that addresses this aspect. Addressing this issue is necessary to understand the complicated process of the liberation of women from their traditional role of a family caregiver to the person responsible for hiring and managing the care provided by migrants. This study describes the experiences and emotions (perception and feelings) of the family member who hires a migrant caregiver for the older relative. In addition, it aims to show the reasons why the women decide to hire migrant women and the positive experiences or difficulties they have with them along the care process.

## 2. Materials and Methods 

In this qualitative study, a phenomenological approach of in-home elder care provided by migrant caregivers was explored to learn the meaning that a human being gives to his/her experience [34,35,36,37,38,39], following the Standards for Reporting Qualitative Research (SRQR standards) [40]:

### 2.1. Researcher Characteristics and Reflexivity

Within the research team, the first author was a nurse with over twenty years of experience as a geriatric and community nurse. Thus, she was very familiar with the phenomenon under study, which takes place in such an intimate space as home. Doubts and questions arose about how the daughters, sons, and helpmates of dependent older people would experience the process of putting their relatives in the hands of migrants to take care of them. This closeness of the researcher to the phenomenon studied, rather than being a limitation, is a significant enrichment for the knowledge of a phenomenon which is scarcely addressed in the scientific literature. In addition, it could contribute reflexivity and depth in the analysis process. 

The multidisciplinary research team included members having experience in working with immigrant women. They were from nursing, sociology, anthropology and health promotion, and worked with an advisory committee. 

### 2.2. Context, Sampling Strategy, and Characteristics of the Participants

We conducted the study among relatives of dependent older people with experience in hiring migrants for home care. The homes of the older people were in different rural villages on the island of Lanzarote. Due to the evolution of the health status of care recipients, participants reported that the hired caregivers were in internal labor regimes.

Participants were selected based on the snowball strategy, which allows us to choose the informants based on the demands of the investigation. For this, the inclusion criteria were people with experience in the phenomenon studied (more than four years of hiring care workers). The main criterion of the expert consultation was related to family members of older people who needed daily life long-term care, hiring migrants in internal labor regimes for such care, or live-in immigrant caregivers. They were chosen based on the information they could offer in terms of providing in-depth knowledge of the reality of the issue [41]. The exclusion criteria were the absence of informed consent from participants and their refusal to cooperate to answer most questions.

Finally, to explore the feelings and meanings associated with this process, the best informants would be the family members themselves, as it would permit hearing first-hand their experiences and emotions about hiring a migrant person to care for their loved one. The attributes of the nine participants are listed in Table 1.

### 2.3. Ethical Considerations Pertaining to Human Subjects

All participants signed a consent form, where they were informed of the purpose and objectives of the research and their participation. They were also informed of their right to abandon the investigation at any time without any consequence. The confidentiality of the participants’ data was ensured during the entire investigation process. The first author coded the personal data and the whole research team worked with the coded identities of the participants.

Prior to the beginning of the study, permission was sought from the pertinent doctorate studies committee of the University of Las Palmas de Gran Canaria.

### 2.4. Data Collection Methods, Instruments, and Techniques to Enhance Trustworthiness

The methods used to gather the necessary information were two in-depth interviews that allowed an insight into the perceptions of family members about their experience with migrants taking care of their dependent relatives at home.

A focus group consisting of seven family members (other than in-depth interviews) allowed us to investigate the attitudes and reactions of the participants. This technique allowed the participants to answer the questions about the group’s interaction, in a dynamic environment in which they felt comfortable and free to speak and comment on their opinions.

The mixture of techniques sought wealth in experiences, contributed by the focus group and the depth of the information provided by the interviews. These interviews were guided to cover similar topics broadly, and participants were asked to illustrate their answers by giving examples. All these offered vast amounts of data for the analysis that ensured the achievement of the research objectives and the information saturation. 

The data collection process was conducted as follows:

The focus group with seven relatives participants was developed in a room assigned by the University of Las Palmas de Gran Canaria in Lanzarote, which maintained adequate and pleasant lighting, acoustic and temperature conditions [42]. The session lasted 120 min. The moderator was the first author along with an assistant trained for this purpose; they also sought to facilitate and encourage informants’ participation.

Two in-depth interviews were conducted three months after the focus group in a session of about 90 min. This had the advantage of going back and refining questions to pursue emerging avenues of inquiry in depth, i.e., the guiding questions for interviews were modified in response to feedback from the first participants in the focus group [43]. All interviews were held at the home of the informants.

All the interviews were audio-recorded, even though interviews and focus groups were conducted using a conversational technique; Box 1 and Box 2 show the script of both methods.

Box 1 **Script for the focus group.**

**Focus Group: Discussion Guide**

**Facilitator’s welcome, introduction, and instructions to the participants**

**Welcome** and thank you for volunteering to take part in this focus group. You have been asked to participate as your point of view and experience, which is important. I appreciate your time.

**Introduction:** This focus group discussion is designed to know your current thoughts and feelings about your experience in hiring foreign caregivers to take care of your dependent relative. The focus group discussion will take no more than two hours. May I tape the discussion to facilitate its recollection?

**Anonymity:** Despite being taped, I would like to assure you that the discussion will be anonymous. The tapes will be kept safely in a locked facility until they are transcribed word for word, then they will be destroyed. The transcribed notes of the focus group will contain no information that would allow individual subjects to be linked to specific statements. You should try to answer and comment as accurately and truthfully as possible. I and the other participants would appreciate it if you would refrain from discussing the comments of other group members outside the focus group. If there are any questions or discussions that you do not wish to answer or participate in, you do not have to do so; however, please try to answer and be as involved as possible.

**Ground rules**

- The most important rule is that only one person speaks at a time.
- There are no right or wrong answers.
- You do not have to speak in any particular order.
- When you do have something to say, please do so. There are many of you in the group and it is important that I obtain the views of each of you.
- You do not have to agree with the views of other people in the group.
- Does anyone have any questions?
- OK, let’s begin.

• **Warm up**

First, I would like everyone to introduce themselves. Can you tell us your name?

• **Introductory question**

I am just going to give you a couple of minutes to think about your experience in hiring migrant people to care for your relatives. Is anyone happy to share her experience?

• **Guiding questions**

- Can you share with us how was your dependent elder and how you experienced the situation of his/her dependency?
- Can you share with us about your responsibility in his/her support?
- Can you talk about the migrant caregivers you have hired? (Sex, age, country of origin…)
- Can you talk about the difficulties you have with the migrant caregivers you have hired?
- Can you talk about the experiences you faced with the migrant caregivers you have hired?
- Could you share episodes of culture shock you faced, if any, while living with the migrant caregivers you have hired?

• **Concluding question**

- Of all the things we’ve discussed today, what would you say are the most important issues you would like to express about your own experience?

• **Conclusion**

- Thank you for participating. This has been a very successful discussion.
- Your opinions will be a valuable asset to the study. We really appreciate your contributions.
- We hope you have found the discussion interesting.
- If there is anything you are unhappy with or wish to complain about, please contact me.
- I would like to remind you that any comments featuring in this report will be anonymous.
- Before you leave, please hand in your completed personal details questionnaire

Please remember to maintain confidentiality of the participating individuals by not disclosing their names.

Box 2 **Script of in-depth interviews.**

**In-Depth Interviews. Addressed Theme Areas and Questions on Each Topic**

**Area: About the dependent elder and how you experienced his reality.**

1. How was your family member's health situation when you decided to hire a person for his/her care?
2. How did your family member receive this care from a person who was not you?
3. How did your family member receive the support from a migrant person?
4. How did you live that situation and how did you feel meanwhile?

**Area: About your responsibility in the care of the older person and how you felt.**

5. Why did you take responsibility for the care of your family member?
6. How was the professional care that your relative could access?
7. Do you share that responsibility with other members of your family?
8. How did you feel in those moments?
9. What are your perceptions, opinions about this situation?

**Area: About hiring migrant caregivers.**

10. Why did you prefer to hire migrants to take care of your family member?
11. How long have you been in this situation?
12. How many caregivers have you hired?
13. Could you tell us the sex, age, country of origin, academic training of the caregivers you have hired?
14. Could you share with me your experience with them, every day with each one, your work with them?
15. Could you comment on the difficulties you had with them?

**Area: About cultural shock.**

16. Could you share what aspects, if any, related to the culture shock you lived with them?

**Area: About your opinion regarding these caregivers.**

17. What did you think, how caretakers were they?

The dialogues were transcribed, whilst respecting the coded identification of the participants. This procedure facilitated the analytical process, because it allowed initial familiarization with the data and served as an “interpretive act” during which meanings were created [35,41,44,45,46]. The analysis of the information obtained from the focus group served as the basis for the elaboration of the in-depth interview script.

In order to guarantee rigor and increase the authenticity of our methodology, both researcher triangulation and data triangulation was used [41]. Analytical rigor was analyzed using multiple encoders. Four team members worked on the analysis and interpretation of the data. The two researchers in the team separately and independently read all the focus transcripts and then developed a coding system. These researchers carried out an inter-subjective validation by consensus of this first codification, which thereafter was used in the thematic content analysis of the data collected subsequently. The third and fourth members of the research team were consulted when needed [47]. All of them were nurses with experience in the subject of study.

### 2.5. Data Analysis

After thoroughly reading the transcribed texts which facilitated the immersion in the data, data reduction was carried out and the coding process identified concepts and discovered the properties and dimensions of the data [35]. Through an inductive method, a first nominal categorization was made. After coding and categorization, analysis and grouping were carried out, followed by the transformation of the data, that is, expression of the data in a new way [35,46,48].

Further, axial coding was developed, in which a system of central categories representative of family discourses was constructed, which directed deeper coding and concluded the analysis process [49,50]. In addition to this, through an interpretative analysis, the candidates for topics and subtopics were created. Using concept maps, the nodes were classified into potential topics and all extracted coded data within the topics were collated. Lastly, the topics were defined and named, identifying the essence of each topic [51]. Finally, the results and conclusions obtained through the contextualization and verification of the findings reached from other studies resulted in a narrative report. The processing and analysis of data were supported by the qualitative data analysis software, Nudist NVivo 10.0 (QSR International, London, UK) [52].

## 3. Results

A total of nine informants participated in this study, and they were all women around 57 years of age. In the majority of the cases, they were the daughters. In addition, none of them, except the wife, lived with the care recipient. Although they did acknowledge carrying out a regime of daily visits, they were more intense when caregivers began to work, and the visits relaxed as the caregiver gained experience and took care of responsibilities.

Eight of the nine participants had a remunerated job, had their own family, were married and had children. The ninth participant was the wife of the care recipient and a housewife, who lived with the care recipient.

The participants reported a long experience in hiring migrant caregivers. All had hired a minimum of four women, while some of them admitted that they had lost count. Hence, it was not possible to make detailed profiles of these caregivers. All the caregivers came from Central and South American countries and none had academic training or formal qualifications to carry out this work. Particularly, until the time of the interviews, a formal employment contract was not mandatory and most of them were in an illegal situation in the country.

We identified three major topics and several subtopics, with respect to each of the research questions raised, as follows: (1) ‘Decisions’ explored the reasons why the relatives decided to hire caregivers, through the following subtopics: ‘I want him/her to be at home, but I cannot care for him/her’; and ‘I want a good caregiver’. (2) ‘Experiences’ comprised two subtopics: ‘adaptation’, with its own subtopics (‘the older person: depends on, needs ... and accepts it’; and ‘the caregiver: learns, respects … and adapts’), and ‘matter of confidence’. (3) ‘How I feel’ presented the emotions and reflections shared by the participants. 

### 3.1. Decisions

The participants could not identify a single reason for hiring a migrant woman but, rather presented a set of motivations that constituted a complex and emotional decision-making process. As discussed below, this complexity was due to the influence of social, cultural, family, personal, and economic factors rooted in Canarian families. Through the simple process of hiring a migrant woman to take care of a family member, the evolution of women in society is exhibited from an extended family where the woman is the caretaker of intangible well-being, to a society in which women try to play a more active role on equal terms with men. 

‘I want him/her to be at home, but I cannot care for him/her’ 

The first decision that the relatives made was to choose the place of residence for the older person. All the participants chose the home, “For me not only is the care important but also that the person can be in his/her own environment" (I1). Consequently, who would take care of the older person was a necessary question to be addressed. The interviewees acknowledged that their personal situation did not allow them to consider the possibility of taking care directly; the words of this daughter clearly showed the pressure to which she was subjected.

“I am the only child, I am working and have three children (…). I was ... overwhelmed, I went from home to work, from work to my mother’s home, (…) I stayed with her all night, returned to my home in the morning half asleep, then to work, and again run to cook something to eat. I was here, leaving, going back, leaving ... well ... it was a chaotic situation for me because no one else could relieve the burden” (I2). 

‘I want a good caregiver’

The participants acknowledged that they would like a good caregiver, "You see that the patient feels loved and protected ... how he is treated by the caregiver because, for example, lovingly turning him over in bed or shaking him sharply are not the same, (...) older people are like glass that can break any time” ... (FG6).

Then, an unexplored path initiated, with new decisions to make, while assuming the consequences of the previous decisions. These were perhaps the most sensitive moments experienced by the participants. They decided to look for migrant caregivers because, as per one of the participants: “The women here (Spaniards) ... will not care for older people” (I2). The time when this phenomenon began on the islands, caregivers were found by word-of-mouth among acquaintances, for example, “There was a family in town who had a Colombian girl, and they told us that they knew about girls from abroad who were interested in working, even living in the house” (I2). 

What did they ask for? The same care as the family themselves provided, at least with affection and respect, if not with love. They did not look for professionals, because they were not professionals either. The participants conceded that they hired these strangers almost without references because, “The most urgent thing is to take care of the person” (FG1) and accepted their lack of professionalism, because in reality, “Who does not know how to care for an older person? They also have their mothers, and they may have children” (I2). Actually, “in a moment of desperation, you will grab whatever to escape from the moment, and then you will continue searching, but when you are at your limit, you can not ... you have to look for anything there is” (I1). 

Although the participants understood that each person was different regardless of where they came from, they accepted that they took the country of origin into account on some occasions, because “Whenever we went to the hospital for the list, they recommended, ’no Colombians’, … because, in the end, we all know each other…” (FG3). In these circumstances, the feelings expressed by the participants were diverse and contradictory. 

### 3.2. Experiences with Migrant Caregivers

#### 3.2.1. Adaptation

However, finding a “good, pretty and cheap” caregiver (if the license is permitted) may not be a guarantee of success. After hiring a caregiver, a new adventure began: a process by which everyone adjusted with each other. One of the sensitive and crucial aspects of this care system is the adaptation of each of the performers involved in the new situation. The relatives played a decisive role in this aspect. As a consequence of a great effort, including continuous supervision and intervention, the relatives tried to achieve harmony, care and safety. 

Reasons for not accepting a caregiver were many, for example the fear of something different: “My mother did not want her at first because of her being black; at night my mother would wake up and be scared” (FG5). There was distrust regarding items being stolen and, in the case of men, a lack of personal comfort, “There are many who do not allow themselves to be bathed, especially by women” (FG7), or “It’s because they are more embarrassed” (FG4). The family members helped the patient navigate this new path, facilitating and working for their well-being. 

‘The caregiver: learns, respects ... and adapts’

The participants recognized that in order to maintain the caregiver’s role, they need to trust in her, which in turn, the caregiver must earn based on having an open attitude toward learning and flexibility in the working conditions, which are both necessary for adapting to the patient’s condition. The participants also considered that the ability and attitude of adaptation of the caregiver were important.

Participants stated that it was necessary to ensure compatibility between the care recipient and the caregiver, so as not, as in this case, “to get home and see them both angry, each by their side” (I1). It was found that the participants helped these women adapt by teaching them to do the work, impart confidence, and even support them in front of the care recipient, “I told my mother we have to get to know her and give her a chance because people cannot be judged by the color of their skin” (FG5). Additionally, in the face of reluctance, “patience is important” (FG9). 

A cultural shock was perceived in issues related to meals: “She never cooked something to eat. If she did, she made their typical food, those fruits with cinnamon ... things like that” (FG3). They also perceived cultural differences while performing tasks, “They have another way of life, another way of being, another character”. To some, the participants had to say “Hey! Look! Turn off the faucet because you’re wasting a lot of water; do not use so much soap, bleach ... everything” (FG4).

Although religious aspects were a cause of problems in some cases, as this participant comments about her caregiver: “The first thing she said was that they had to remove the picture of the Sacred Heart of Jesus that my mother had in her room” (FG2). It was not usual, and coexistence was usually imposed, for which mutual respect was key, “She has not tried to impose on me, nor do I try to impose...” (FG5).

The caregivers hired by the participants did not have training for this work: “She did not know much because some of the things she did, she did not know how they were, but well …” (FG7). Therefore, the participants were responsible for training them and teaching them everything necessary associated with the care. The participants valued the predisposition of the caregiver, for example: “To me, it is important that they desire to learn because if I do not know everything, I can learn from you” (FG5). While trying to teach, the adaptation of the caregiver to the home and to the care recipient was encouraged. The participants contemplated that to help the caregiver adapt, one must understand that “When you come to a place with different customs, with different habits, until you adapt, you do what you think you should do, although probably it does not match others’ expectations…”(FG3). 

#### 3.2.2. Matter of confidence

The participants conceded that trusting the caregiver was not something that happened all of a sudden; rather, it was a gradual process. Important characteristics for trusting the caregiver included their attitude, for example the desire to learn, as well as showing flexibility and availability to changing schedules, which was a highly valued feature, because the care needs of the care recipient continuously evolved, making schedule modifications necessary: “She has her free days, but if anything arises, we need her to be available” (I1). Accepting changes in the working conditions previously agreed upon was also an important characteristic: “I said, what interests you most, changing the days, or pay for those days? Because of course, you see that she needs the money” (I2). Due to the lack of trained caregivers, the participants positively valued the desire of the caregiver to learn. This comment from an interviewee shows her understanding of the situation and how happy she was with her caregiver: “She has even learned gardening. My mother has taught her how to plant, to look over ... and she by her own initiative is already going to plant pumpkins ... although my mother always has complaints” (I1).

From the dialogue of the participants, there were three different ways of showing confidence in the caregivers: 

Delegating more responsibilities and tasks to them, while watching less and less over them. 

Supporting and helping them in their daily work: “I always told my caregivers that we were going to help them in everything we could” (I2). 

And treating them as part of the family: “I told her, you are like my sister, you are here, you have my father who is also fatherly to you because he gives you advice just like me” (I2).

However, the participants also came across a few negative experiences after years with caregivers.

A strong personality of the caregiver, i.e., those who were inflexible and believed their norms or customs were being challenged, was a primary reason for dismissal, or for the caregiver deciding to leave in many cases. One participant shared what happened when the caregiver did not want to do things the way she had been asked: “The next day at 8 o’clock in the morning, she was sitting on the terrace with her suitcase. She said she was leaving and it was up to me if I wanted to pay her...” (FG4).

Attempts by caregivers to manipulate through emotional blackmail for economic benefits were also reported, such as sending extra remittances to their home countries: “Oh, why do not you pay me for the package I’m going to send to my country?”, or “Oh, I sent all the money over there” (FG5). Another problem was that the caregivers sometimes made intercontinental telephone calls without paying for them: “She called up every day for a month and she left before the bill came. We found out because they cut the phone off” (I2). Only one of the participants reported a more serious case: “When a civil guard came to the house to look for her because she robbed us. Thank God nothing happened to my mother” (FG2). The participants commented that good experiences with caregivers enhanced trust in them, while bad experiences caused distrust, although not an absolute rejection of these people.

### 3.3. How I Feel

In the process of deciding to hire a migrant caregiver, the participants recognized that they felt a plethora of emotions: “You feel everything, insecurity because you do not know if you are going to find one, it is an internal struggle because you have to maintain your status and that of your children, and helplessness because there are no nursing homes …” (I1).

In general, some participants acknowledged that they appreciated the help because “If you think about it, the job they do is actually not adequately paid for, you know? Really, it’s 24 h job…” (FG3). They also commented that it was a very hard process: “You know? It is difficult, for my mother, as well as, for family members” (FG3), and “For the family, it is a great sacrifice to maintain this situation because you have to be on the side of both parties” (I2). Nevertheless, the participants did not stop trusting these women, despite their previous negative experiences: “I think that this can hardly be done because you have a person living in your house with you, hand to hand, side by side, eating what is yours, participating in everything that is yours …” (FG6).

For many, the journey has been very long: “Since 1999 they have been taking care of her; 14 years since we had our first contact with a person who came to our house, and that at that time was the beginning …” (FG2).

## 4. Discussion

The idea of home care for dependent older individuals, carried out by female migrants, has become a frequent reality globally [5,17,18]. However, the perspectives and experiences of relatives who hire these women are barely addressed in the literature. These family members are one of the great pillars on which this system of care is based [19,29,30,53], which is in accordance with the family values of the older person who is being cared for at his/her own residence [13,33,47,54].

The typical profile of the participants included a middle-aged woman who is, in accordance with the literature, an available member of the care recipient’s family [55]. According to the sample size indications for phenomenological studies and homogeneous samples [45,56,57], nine participants with long and deep experience in the subject contributed to the credibility and conformability of the methodology and presented a new way of viewing migrant caregivers in the current socio-cultural context; the perspective of a daughter or a wife who had to hire and administer a caregiver, an aspect for which she was not prepared and which she has had to face due to the cultural, socioeconomic, and familial circumstances [9,13].

‘I cannot take care of them.’ The study participants recognized that they were not able to take care of the family member directly, due to the responsibilities they shared as female caregivers and mothers. This hints at the fact that Canarian women are similar to those all over the world, experiencing the same challenges faced by female generations for decades, while also going through the so-called crisis of care, among others [7,54,58,59,60,61]; i.e., the families who care for loved ones are experiencing intolerable strain [62].

‘Reasons for hiring a migrant.’ The selection of migrant caregivers was of a conjunctural nature, where economic reasons were more important [43]. The lower salaries anticipated by these people is a good reason to hire them, which is in concordance with other studies [17,33]. The global economic crisis manifested itself most severely in social services, mainly affecting the economic capacities of the individuals [4,7,63]. This, in turn, has hindered access to professional care; therefore, the interviewees sought for a solution by hiring other women migrants, who came to overcome financial difficulties, although they recognized the importance of pay and working conditions [64,65]. These women justified their selection as a form of adaptation to circumstances, which allowed them to meet the care needs, an observation that is consistent with that of Moreno-Colom et al. [31]. The condition that many caregivers were illegal is worth noting, as this factor did matter, since it was not required to formalize a legal contract. On the other hand, legal contracts for helpers were not compulsory in Spain until the regulatory law came into force in 2011 [66,67]. Another important reason for hiring immigrant caregivers found in this study is the desire of the participants to be replaced in their role as daughters, in the sense of ensuring that the caregiver provided care and dedication to the dependent elder. This is consistent with the desire to alleviate the feelings of guilt that are caused by not taking care of their family member directly [26,28,68]. 

‘Their experiences.’ The report of their experiences has helped understand the efforts made by these women to maintain the quality of care provided by them. They helped their dependents accept their situation (i.e., dependence on others), as well as their need to receive care and also accept the new caregiver, thus continuing with the family values of caring for elders in the family, which is commonly seen in many cultures around the world [69]. 

The family members supported the caregivers by training them on the care required and resolving conflicts resulting from cultural shock, which inevitably presents itself in this type of a relationship, and on the subject of getting along with the older person [70]. This mediation played by the family member between the caregiver and themselves is also found in other studies, though only from the point of view of the migrant caregiver herself and not the relative [71].

Trust is a key element in the theory of this type of care, although literature is scarce with regards to this particular issue. It is necessary for the family members to trust the caregiver, for her employment to be continued. As long as there is a lack of trust, the family would continue looking for a person worthy of that trust. One aspect that has fostered trust is the attitude of the caregiver. In a situation in which the caregiver has little knowledge about her work, her openness to learning, conciliatory nature, patience with the care recipient and acceptance of flexible conditions depending on the evolution of the older person’s situation is valued. The participants showed confidence by giving the caregiver more responsibility, providing less supervision, making them feel as though they are a part of the family [72]. This subjective interpretation has been described in previous studies [73] and allows for the extension of membership to a broad range of people not traditionally considered family, such as close friends, or caregivers without formal family ties. An aspect lacking in the literature corresponds to the negative experiences that have been stated, such as emotional blackmail to obtain extra economic benefits, deception, and robberies. 

‘Emotions or Feelings.’ As daughters of sick people whose well-being they feel responsible for, women family caregivers recognized that they feel a complex set of emotions, such as the burden of responsibility to care for themselves and the entire family and the desperation to find a solution for fulfilling that responsibility [9,13,16,53]. They also felt fearful as to what might happen to them. This process seems difficult and, in the long run, tiring. Some of these emotions have been found in the family members. They may be related not only to the socio-cultural aspects of female role, but also to the sense of compassion when empathizing with their loved one suffering and their desire to cope appropriately so as to solve the situation [74]. An Asian study examined the dynamics of formal and informal caregiving of older people within the family sphere, where family caregivers identified financial, physical, emotional and psychological stresses in their roles as caregivers. They described tension about the adjustment of the migrant domestic caregiver to the household routine and the demands of the care recipient, and lack of appreciation from the care recipient to the caregiver. The juxtaposition of emotional closeness with the sense of filial obligation to care resulted in the expression of ‘no choice’ [75].

### Limitations and Future Directions

The authors acknowledge the limitations of the purposive and convenience sampling techniques applied in the research, such as sampling bias and the lack of representativeness of the findings. We might thus have introduced bias by selecting relatives who were most likely to be able to take part in the study. The majority of relatives were daughters, whose needs, feelings and expectations may differ from other relatives who care for an elder in the family [47]. One of the major limitations of this idea is the very own nature of the work: at home, a private space, and difficult to access. However, given the difficulties in recruiting participants in this study, it would be interesting to carry out more research on this subject, so as to deepen the knowledge about the perception of these families and the migrant caregivers. It would have been useful to have more detail about individual migrant caregivers (country, age, if received adequate training and development, or remuneration and support). On the other hand, although semi-structured interviewed were useful, they might have also resulted in certain aspects of the relatives’ experiences remaining unexplored, as a result of preconceived notions of important areas warranting discussion. Sometimes, the members of the research team discussed the themes and came to a consensus. Or, finally, the participants may have framed their responses to present themselves favorably to the researchers (social desirability) [76]. The significance of this study is the extent to which findings can be generalized, beyond the setting in which they were generated. This is a very common situation across the globe, where a comprehensive suite of policies that focus on the needs of family caregivers of older relatives is lacking.

## 5. Conclusions

Family members exhibited their trust by delegating more responsibilities to the caregivers with less supervision of their work. Additionally, the participants treated the caregiver as a part of the family, which shows their affection toward the caregiver, to the extent that some caregivers continued to live with the family members of the dependent older person even after his/her death. 

The family seeks more than just the professional care from the caregiver; they seek the same attention and affection which they give to their family. In this way, the study participants became the caregivers’ managers, since they presumed the responsibility of care and trained and helped the caregivers perform these tasks. The situation of dependence reveals how the moral values make women bear the obligation to respond to the need for care, and that they experience conflict with their own situation: working outside the home, caring for the rest of the family, and living their own life. All the study participants showed great affection and desire for the well-being of their loved ones. 

## Figures and Tables

**Table 1:** Characteristics of the Participants.

Identification Family Member	Sex	Age	Relationship with the Elderly Person	Years of Hiring Migrants	Live with Him/Her
I 1	Female	60	Daughter	5	No
I 2	Female	62	Daughter	6	No
FG1	Female	61	Daughter	5	No
FG2	Female	53	Daughter	14	No
FG3	Female	45	Daughter	7	No
FG4	Female	72	Wife	15	Yes
FG5	Female	50	Daughter	8	No
FG6	Female	53	Daughter	9	No
FG7	Female	58	Daughter	10	No

I = interview FG = Focus Group.
